# Requirement of dual stimulation by homologous recombinant IL-2 and recombinant IL-12 for the *in vitro* production of interferon gamma by canine peripheral blood mononuclear cells

**DOI:** 10.1186/1756-0500-7-460

**Published:** 2014-07-18

**Authors:** Andrea Mendes Pereira, Cristiane Garboggini Melo de Pinheiro, Lenita Ramires dos Santos, Naiara Carvalho Teixeira, Yung-Fu Chang, Lain Carlos Pontes-de-Carvalho, Geraldo Gileno de Sá Oliveira

**Affiliations:** 1 Laboratório de Patologia e Bio-Intervenção, Centro de Pesquisas Gonçalo Moniz, Fundação Oswaldo Cruz, Rua Waldemar Falcão, No. 121, Candeal, Salvador, Bahia, Brazil; 2 Department of Population Medicine and Diagnostic Sciences, College of Veterinary Medicine, Cornell University, Ithaca, NY, USA; 3 Instituto Nacional de Ciência e Tecnologia de Doenças Tropicais (INCT-DT), Salvador, Bahia, Brazil

**Keywords:** Interleukin 2, Interleukin 12, Cellular immune response, Dog

## Abstract

**Background:**

Very few studies have been carried out so far aiming at modulating cellular immune responses in dogs. In this study, we evaluated the ability of recombinant canine IL-2 (rcaIL-2) and IL-12, in the form of a single-chain fusion protein (rsccaIL-12), to stimulate peripheral blood mononuclear cells (PBMC) of healthy mongrel dogs.

**Results:**

Recombinant canine IL-2 purified from *Escherichia coli* or present in the supernatant of COS-7 cells transfected with pcDNA3.1-caIL-2 (COS-7 caIL-2 supernatant) was able to induce proliferation of CTLL-2 cells, thus showing their functional activity. In addition, purified rcaIL-2 and COS-7 caIL-2 supernatant stimulated resting canine PBMC proliferation to a level higher than baseline level. Neither COS-7 sccaIL-12 supernatant nor COS-7 caIL-2 supernatant alone was able to induce significant production of interferon gamma by resting PBMC. However, COS-7 sccaIL-12 supernatant in combination with COS-7 caIL-2 supernatant induced production of IFN-γ by those cells.

**Conclusions:**

The data shown herein suggest that the combination of canine recombinant IL-12 and IL-2 can be useful to promote cellular immune responses in dogs.

## Background

Cytokines are proteins or glycoproteins produced mainly by cells of the immune system. They participate in several different biological processes, including the homeostasis of the immune system and the generation of immune responses [1,2]. To exert their biological activities, cytokines bind to specific receptors on the cytoplasmic membrane of the target cells and activate intracellular signaling pathways that result in the generation of transcription factors and expression of several genes [3]. Being involved in the initiation, effector mechanisms and regulation of inflammation, as well as in the immune responses, cytokines contribute both to the development and control of many different diseases, including the infectious, parasitic, allergic, autoimmune and neoplastic diseases. Therefore, substances that interfere with cytokine signaling pathways, both agonists and antagonists, may be useful for the prophylaxis or therapy of a great number of diseases.

There is a large amount of data showing that the manipulation of the immune system by interference in cytokine signaling changes the outcome of various diseases in the murine model. However, no studies comparable to those carried out in mice that could encourage the development of new therapeutic approaches for canine diseases have been reported in the dog.

The control of infection by intracellular pathogens can be initiated by stimulation of natural killer (NK) cells and be established by cellular immune responses involving CD4^+^ T cells, activation of macrophages, and CD8^+^ T cells [4]. Little is known, however, about conditions that promote cellular immune responses in dogs.

Interleukin-2 (IL-2) was the first cytokine to be tested in a purified state or cloned [5,6]. IL-2 is produced mainly by CD4^+^ T cells and CD8^+^ T cells activated by antigen [7,8]. It performs various activities in the immune system, including promotion of differentiation of CD4^+^ T helper-1 (Th1) and -2 (Th2) cells, maintenance of CD4^+^ regulatory T (Treg) cells and optimization of the expansion of CD8^+^ effector T cells and CD8^+^ memory T cells [9].

IL-12 is a heterodimeric cytokine produced by antigen-presenting cells (dendritic cells, macrophages and B cells) that, alone or synergistically with other factors, e.g., IL-2 or mitogens, displays the ability to stimulate the production of interferon gamma (IFN-γ) in resting or activated NK and T cells [10]. IL-12 is also able to promote the cytotoxic activity and proliferation of NK and T cells [10]. Furthermore, IL-12 contributes to the generation of Th1 lymphocytes [11], which are required for controlling infections by intracellular pathogens.

Although several authors have reported some consequences of the exposure of recombinant human IL-2 (rhuIL-2) or IL-12 (rhuIL-12) on canine systems *in vitro* and *in vivo *[12-18]*,* reports on the exposure of canine cells to recombinant canine IL-2 (rcaIL-2) do not exist and to recombinant canine IL-12 (rcaIL-12) still provide insufficient knowledge to allow for the exploitation of the full potential of this cytokine in the manipulation the immune system of dogs [19-23]. The administration of heterologous cytokines in animals, even when the homology between the donor and receptor species is high, may result in the production of specific antibodies with the potential ability to prevent the action of long-lasting treatments [24-26]. Therefore, in order to manipulate the canine immune system for long-term, it would be better to use homologous instead of heterologous cytokines.

Since IL-2 synergizes with IL-12 to promote NK and T cell proliferation, cytotoxic activity, and IFN-γ production [27-30], it would be interesting to study the effects of the combination of these two cytokines in dogs. In this paper, cloning of canine IL-2, expression of rcaIL-2 and rcaIL-12 and the ability of these cytokines to stimulate canine PBMC are reported.

## Methods

### Cloning, production and purification of canine IL-2

The cDNA of canine IL-2 was inserted into a plasmid either for expression in *Escherichia coli* or in mammalian cells, using conventional methods. Briefly, the cDNA encoding the mature protein was amplified by PCR from a pCRII-2-caIL-2 template (kindly donated by Dunham, Department of Veterinary Pathology, University of Glasgow Veterinary School, United Kingdom) with specific primers (forward 5′-CGCGGATCCGGCACCTATTACTTCAAG and reverse 5′-CCGGAATTCTCAAGTCAGTGTTGAG) and digested. The digestion product was inserted into pRSET (Invitrogen Corporation, Carlsbad, EUA) using the BamHI/EcoRI site, generating the pRSET-caIL-2 construction.

The cDNA encoding the whole IL-2 protein was amplified by RT-PCR from canine PBMC, after their stimulation with 10 μg/mL concanavalin A (Con A, Sigma-Aldrich, St. Louis, USA) for 24 h at 37°C in a 5% CO_2_ atmosphere, using specific primers (forward 5′-CGCGGATCCAAGCCACCATGGGCAAAATGCAACTCTTGTCTTGC and reverse 5′-CCGGAATTCTCAAGTCAGTGTTGAGAAGATGC). The cDNA was digested and inserted into the BamHI/EcoRI site of the pcDNA3.1 plasmid (Invitrogen), resulting in the construction pcDNA3.1-caIL-2.

The plasmid DNA molecules with the inserts were sequenced in an ABI3100 automatic sequencer (Applied Biosystems, Foster City, CA, USA).

The production of rcaIL-2 was performed in BL21 strain (DE3)pLysS *E. coli* (Invitrogen) transformed with pRSET-caIL-2 following the manufacturer’s recommendations. The bacterial cell pellet, obtained by centrifugation after inducing the protein expression for 5 h with 1 mM isopropyl-β-D-thiogalactoside (IPTG), was suspended in 50 mM Tris, pH 7.6, containing 1% Triton X-100 (v/v) and 1 mg/mL lysozyme. The suspension was then subjected to ultrasonication. The inclusion bodies were collected by centrifugation, solubilized in 20 mM Na_2_HPO_4_, 500 mM NaCl, 10 mM imidazole and 8 M urea, pH 7.4, and purified in a nickel-Sepharose affinity column (Amersham Biosciences, Uppsalla, Sweden), following the manufacturer’s recommendations. The purified protein was refolded using the dialysis method described by Guisez and collaborators [31] and the protein concentration was determined by Bradford’s method.

The purity of the affinity-purified IL-2 was assessed by polyacrylamide gel electrophoresis in the presence of sodium dodecyl sulfate (SDS- PAGE) and by Western blot using anti-histidine monoclonal antibody (Invitrogen). His-tagged recombinant green fluorescent protein (EGFP), produced in BL21(DE3)pLysS *E. coli*, and chromatographically affinity purified, was used as a positive control. To promote stability of the purified protein, bovine serum albumin was added to achieve a final concentration of 1 mg/mL [31]. Aliquots were prepared and stored at -20°C until use.

### Expression of canine IL-2 and IL-12 in eukaryotic cells

Recombinant canine IL-2 and IL-12 were separately expressed into culture supernatants of COS-7 cells transfected with pcDNA3.1-caIL-2 or pcDNA3.1-sccaIL-12, using the method previously described [20]. The latter encodes IL-12 protein in the form of a single chain fusion protein [20]. Supernant of COS-7 cells transfected for 48 h with pcDNA3.1-caIL-2 (COS-7 caIL-2 SN), pcDNA3.1-sccaIL-12 (COS-7 sccaIL-IL-12 SN) or pcDNA3.1 without insert (COS-7 SN-negative control) were collected and stored at -20°C until use.

### Assessment of biological activity of recombinant canine IL-2 (rcaIL-2)

The biological activity of rcaIL-2 produced in *E. coli* or COS-7 cells was evaluated using CTLL-2 cells (TIB-214 cells, American Type Culture Collection, ATCC, USA), essentially as previously described [32]. Briefly, in triplicate wells of 96-well flat bottom microtiter plates (Corning Incorporated), 10^4^ CTLL-2 cells/well in RPMI 1640 culture medium supplemented with 2 mM of sodium pyruvate, 2 mM of L-glutamine and 10% fetal calf serum (supplemented RPMI 1640) were cultured with: 1) rcaIL-2 purified from *E. coli* at final concentrations between 9 pg/mL and 700 ng/mL; 2) COS-7 caIL-2 SN or SN COS-7-negative control at final concentrations between 0.39 and 12.5%; 3) supernant of rat splenocytes stimulated with Con A), the so-called T-STIM (BD Biosciences, Franklin Lakes, NJ, USA) at 10% (positive control); and 4) supplemented RPMI 1640 culture medium. The plates were incubated for 24 h at 37°C in a humidified atmosphere of 5% CO_2_. Thirty μL of supplemented RPMI medium containing 1 μCi of ^3^H-thymidine were then added to each well. After a further 24 h period of incubation, the cells were collected onto glass fiber membranes (Packard , Meriden, USA) and analyzed in a Matrix 9600 Direct Beta Counter (Packard), that generated counts per minute (CPM) values related with beta particle emission.

### Canine peripheral blood mononuclear cells (PBMC)

Eight healthy adult mongrel dogs, 4 males and 4 females, were used. The animals were kept in the Gonçalo Moniz Research Center kennel with access to water and food *ad libitum*. All experiments were performed in accordance with the guidelines of the Oswaldo Cruz Foundation experimental animal use (Gonçalo Moniz Research Center Ethical Committee Experimental Licence Protocol 040-CPqGM). Twenty-five mL of blood from each dog were collected from the cephalic vein with a heparinized syringe (Heparin, Eurofarma, São Paulo, Brazil). Blood samples, diluted 1:2 in Hanks solution (HBSS, Sigma-Aldrich) buffered with 10 mM HEPES (Invitrogen), pH 7.0, were placed onto Fycoll-Hypaque (Sigma-Aldrich) and centrifuged for 35 minutes at 800 × g, at 20°C. The mononuclear cells were collected, washed twice with buffered HBSS and suspended in RPMI 1640 supplemented with 10 mM HEPES, 10 μM 2-mercaptoethanol (complete RPMI 1640) and 50 μg/mL gentamicin sulfate (New Farma Ltda., Anapolis, Brazil) to 2 × 10^6^/mL.

### Peripheral blood lymphocyte proliferation after stimulation with rcaIL-2 or COS-7 caIL-2 SN

One hundred μL suspensions of 2 ×10^5^ PBMC of 6 dogs were added in triplicates to wells of 96-well flat-bottom microtiter plates. To each well it was also added a 100 μL volume of: 1) rcaIL-2 produced in *E. coli,* to reach a final concentration of 50 ng/mL; 2) complete RPMI 1640; 3) COS-7 caIL-2 SN or COS-7 SN-negative control, to achieve a final concentration of 0.5, 5 and 50%. The plates were incubated at 37°C in a humidified atmosphere of 5% CO_2_ for 4, 6, 8, 10, 12 or 14 days. Eighteen hours before harvesting the cells, 30 μL of RPMI 1640 with 1 μL Ci ^3^H-thymidine were added to each well. The cells were collected and evaluated as described above.

### Detection of interferon gamma in cultures of PBMC stimulated with IL-12 and/or IL -2

One hundred μL of a suspension of 2 ×10^5^ PBMC were added to each well of 96-well flat-bottom microtiter plates. It was also added to the same wells, in triplicate, 100 μL volumes of: 1) complete RPMI 1640; 2) Con A at 5 μg/mL; 3) COS-7 caIL-2 SN at 25%; 4) COS-7 sccaIL-12 SN at 0.025, 0.5 or 10%; or 5) COS-7 sccaIL-12 SN at 0.025, 0.5 or 10% combined with COS-7 caIL-2 SN at 0.02, 1, 5, or 25%. The plates were incubated for 48 h at 37°C in a humidified atmosphere with 5% CO_2_. The culture supernatants were removed and stored at -20°C until use. The concentration of interferon gamma (IFN-γ) was measured in the supernatant by capture ELISA, using reagents from R&D Systems (Minneapolis, USA) and following the manufacturer’s recommendations. Briefly, 96-well high-binding microtiter plates (Corning Incorporated Life Sciences) were coated with 100 μL/well of anti-canine IFN-γ at 2 μg/mL and blocked with 300 μL/well of 1% bovine serum albumin (BSA), 5% sucrose, and 0.05% NaN_3_ in PBS. One hundred μL of the test samples were applied in duplicates to the wells. For the elaboration of a calibration curve, recombinant canine IFN-γ was used in duplicate at concentrations between 62.5 pg/mL to 16 ng/mL. The following reagents were successively applied: a) anti-canine IFN-γ - biotin conjugate at 100 ng/mL; 2) streptavidin-peroxidase conjugate diluted 1:200; and 3) tetramethylbenzidine substrate (Sigma- Aldrich). The reaction was stopped by adding 50 μL/well of 1 M H_2_SO_4_ and the plates were analyzed at 450 nm in an Emax Precison Microplate Reader (Molecular Devices Corporation, Sunnyvale, USA). The concentration of canine IFN-γ present in each sample was estimated using the Softmax 3.0 program, corrected for the dilution factor when needed.

### Statistical analysis

For the lymphoproliferation assays, the medians of CPM were compared using the non-parametric Friedman test followed by the Dunn’s test, since CPM data corresponding to thymidine incorporation generally does not have a normal distribution. The values for IFN-γ concentration were compared by ANOVA and Dunnett’s post-test. Values of p < 0.05 were considered significant.

## Results

### Cloning of canine IL-2 and production in *E. coli*

The DNA sequencing of pRSET-caIL-2 construction insert showed two nucleotide mutations at positions 187 (T= > C) and 243 (A= > G), in comparison with the cDNA encoding the full-length protein previously described by Dunham and collaborators [33], resulting in a substitution of a phenylalanine for a leucine (F = > L) residue. This corresponds to a change of the 43rd amino acid residue in the mature protein, and the resulting recombinant protein was called rIL-2-F43L.

The DNA sequencing of pcDNA3.1-caIL-2 construction insert revealed a complete identity with cDNA encoding the full-length canine IL-2, described by Dunham and collaborators [33], with the exception of the nucleotide at position 4 (T= > G), deliberately changed to introduce the consensus eukaryotic ribosome binding site sequence [34].

**Figure 1:**
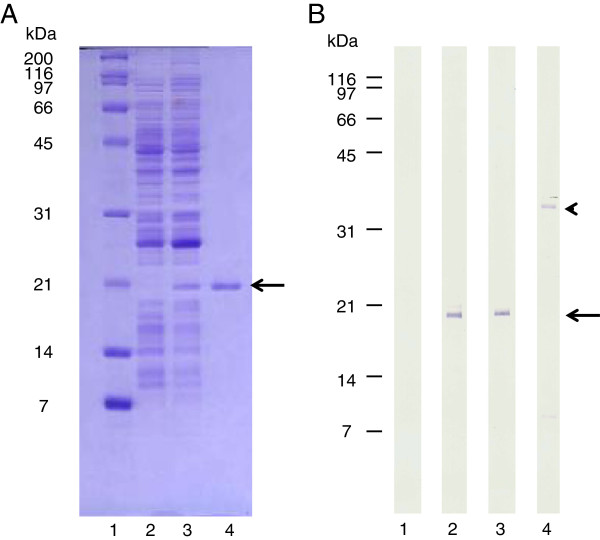
**Evaluation of rcaIL-2 expression and affinity purification by SDS-PAGE and Western blot. A**. Samples analysed in 15% polyacrilamide gel stained with Commassie blue. Lane 1, Molecular weight markers; Lane 2, *E. coli* BL21(DE3)pLysS-pRSET without insert; Lane 3, BL21(DE3)pLysS-pRSET-caIL-2; Lane 4, affinity purified rcaIL-2-F43L. **B**. Samples evaluated by Western blot developed with an anti-His tag monoclonal antibody conjugated with alkaline phosphatase. Lane 1, *E. coli* BL21(DE3)pLysS-pRSET without insert; Lane 2, BL21(DE3)pLysS-pRSET-caIL-2; Lane 3, affinity purified rcaIL-2-F43L; and Lane 4, affinity purified His-tagged enhanced green fluorescent protein (EGFP, positive control). Arrows and arrow head indicate rcaIL-2F43L and EGFP bands, respectively.

Canine rIL-2-F43L with an N-terminus histidine tag, produced in *E. coli* and purified by affinity chromatography, displayed a single band with a molecular weight of 19.3 kDa in SDS-PAGE and Western blot analysis (Figure 1).

### Biological activity of cloned canine IL-2

**Figure 2:**
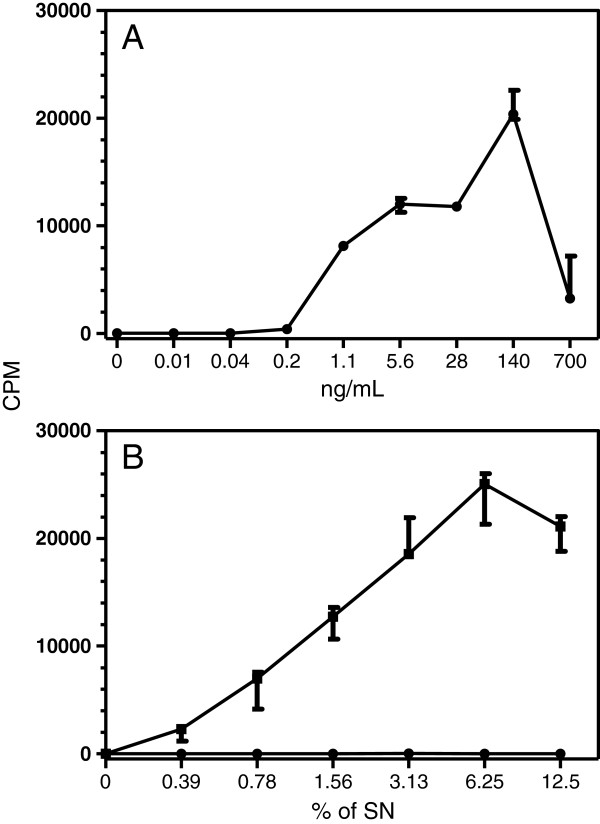
**Evaluation of CTLL-2 proliferation following stimulation with recombinant canine IL-2 produced by *E. coli *or COS-7 cells**. CTLL-2 cells were cultured for 48 h with: **(A)** different concentrations of affinity purified rcaIL-2-F43L produced in *E. coli* or **(B)** different concentrations of supernatant of COS-7 cells transfected with pcDNA3.1 without insert (circles) or pcDNA3.1-caIL-2 (squares). The cells were pulsed with 3H-thymidine for 24 h. The data represent the median of CPM of triplicates and the bars the 25 and 75 percentiles.

To assess whether the canine rcaIL-2-F43L subjected to refolding or the COS-7 caIL-2 SN would present biological activity, the CTLL-2 cell proliferation assay was performed, and median (M) values of CPM, that are related to thymidine incorporation, were used to analyze the results. CTLL-2 cells cultured in supplemented RPMI 1640 medium alone (negative control) or stimulated with 10% T-STIM (positive control) showed no (M = 14.0) or high (M = 73,166) proliferation activity, respectively. CTLL-2 cells cultured with rcaIL-2-F43L in concentrations ranging from 0.22 ng/mL (M = 413) to 700 ng/mL (M = 3,239) displayed higher proliferative response than cells incubated with supplemented RPMI 1640 alone (Figure 2A). However, the proliferation peak was reached when rcaIL-2-F43L was used at 140 ng/mL (M = 20,380) (Figure 2A).

COS-7 cells caIL-2 SN also induced proliferation of CTLL-2 cells when used at concentrations ranging from 0.39% (M = 2,272) to 12.5% (M = 21,110), as compared with cells incubated with COS-7 SN-negative control (highest value of M = 10) (Figure 2B). Interestingly, the proliferation peak was reached with COS-7 caIL-2 SN at 6.25% (M = 25,068) (Figure 2B). These data indicated that both the recombinant protein produced in *E. coli* and refolded and the one produced in COS-7 cells is functionally active.

### Proliferation of resting PBMC stimulated with cloned canine IL-2

**Figure 3:**
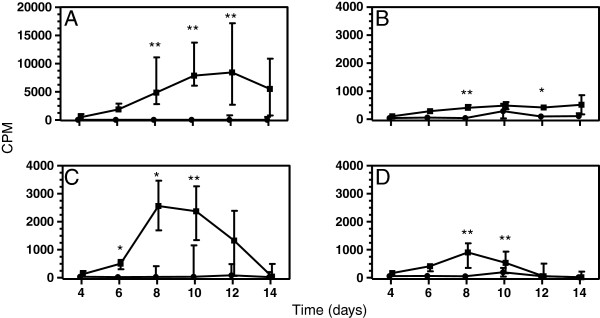
**Evaluation of proliferation of canine peripheral blood mononuclear cells (PBMC) stimulated with recombinant canine IL-2 produced by *E. coli *or COS-7 cells**. PBMC of 6 adult mongrel dogs were cultured for 4 to 14 days with: **(A)** affinity purified rcaIL-2-F43L produced in *E. coli* at 50 ng/mL (squares) or excipient (circles); **(B, C, and D)** supernatant of COS-7 cells transfected with either pcDNA3.1 without insert (circles) or pcDNA3.1-caIL-2 (squares) at 0.5% **(B)**, 5% **(C)** or 50% **(D)**. The cells were pulsed with 3H-thymidine for 18 h. The data represent the median of the CPM obtained for the six animals and the bars the 25 and 75 percentiles. *p < 0.05, **p < 0.01; Dunn’s post-test (p < 0.001, Friedman’s test).

To determine if the rcaIL-2 produced in *E. coli* or the COS-7 caIL-2 SN would promote proliferation of resting lymphocytes, PBMC from six healthy dogs were cultured for 4 to 14 days with rcaIL-2-F43L at 50 ng/mL or COS-7 caIL-2 SN at 0.5, 5 or 50% and thymidine incorporation was measured. Whereas PBMC cultured in complete RPMI medium alone, used to determine the basal proliferation, presented low incorporation of thymidine, with CPM values ranging from M = 9 to M = 31 along the time points assessed, cells cultured with rcaIL-2-F43L displayed significantly higher CPM values at days 8 (M = 3,542), 10 (M = 7,854) and 12 (M = 11,928), corresponding to an increase of up to 918 times above the basal levels (p < 0.05, Dunn’s test; Figure 3A). Moreover, the PBMC incubated with COS-7 caIL-2 SN showed significantly higher thymidine incorporations when tested at 0.5% for 8 days (M = 237 vs 14 for the control) and 12 days (262 vs 10) , 5% for 6 days (388 vs 22), 8 days (2,161 vs 26) and 10 days (2,375 vs 28), and 50% for 8 days (780 vs 26) and 10 days (735 vs 32), reaching up to an 87-fold increase, as compared with the COS-7 SN-negative control (Dunn’s test p <0.05, Figure 3B, C and D).

### Production of IFN-γ by canine PBMC stimulated with recombinant homologous IL-12 and/or IL-2

**Figure 4:**
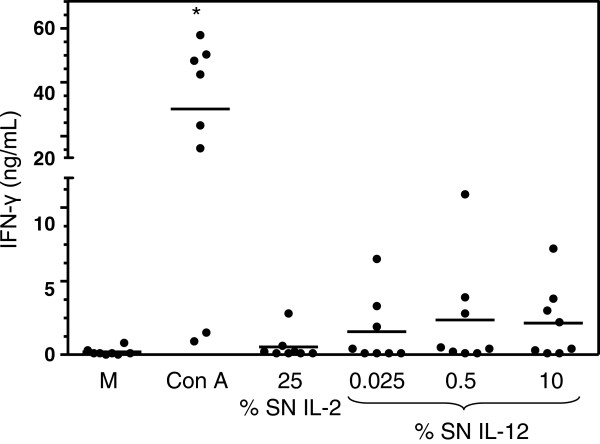
**Interferon gamma (IFN-γ) production by peripheral blood mononuclear cells (PBMC) stimulated with recombinant canine IL-2 or IL-12 produced by COS-7 cells**. PBMC of 8 adult mongrel dogs were cultured for 48 h with: complete RPMI 1640 medium alone (M), concanavalin A at 5 mg/mL, supernatant of COS-7 cells transfected with pcDNA3.1-caIL-2 (SN IL-2) at 25% or supernatant of COS-7 cells transfected with pcDNA3.1-sccaIL-12 (SN IL-12) at 0.025, 0.5 or 10%. IFN-γ was assessed in the PBMC supernatants by capture ELISA. Data represent average of duplicates and bars the means obtained for the eight animals. *p < 0.01, Dunnett’s test (p < 0.0001, repeated measures ANOVA).

**Figure 5:**
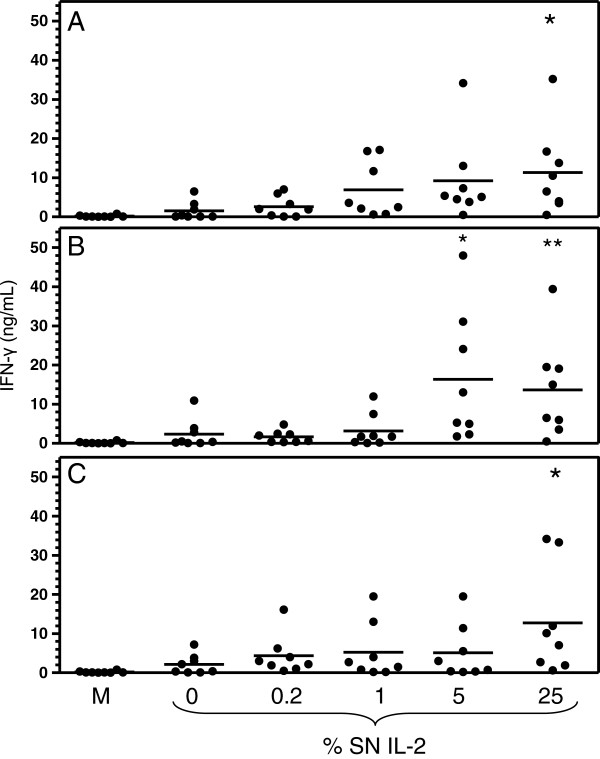
**Interferon gamma (IFN-γ) production by peripheral blood mononuclear cells (PBMC) stimulated with recombinant canine IL-2 and/or IL-12 produced by COS-7 cells**. PBMC of 8 adult mongrel dogs were cultured for 48 h with: a) complete RPMI 1640 medium alone (M), b) supernatant of COS-7 cells transfected with pcDNA3.1-caIL-2 (SN IL-2) at 0, 0.2, 1, 5 or 25% in combination with supernatant of COS-7 cells transfected with pcDNA3.1-sccaIL-12 at 0.025 **(A)**, 0.5 **(B)** or 10% **(C)**. IFN-γ was assessed in the PBMC supernatants by capture ELISA. Data represent average of duplicates and bars the means obtained for the eight animals. *p < 0.05, **p < 0.01; Dunnett’s test (p < 0.0001, repeated measures ANOVA).

To evaluate the ability of the recombinant IL-12 and/or IL-2 to stimulate the production of IFN-γ by canine cells, PBMCs from 8 healthy dogs were cultured with different concentrations of COS-7 sccaIL-12 SN combined or not with various concentrations of COS-7 caIL-2 SN for 48 h. The concentration of IFN-γ (mean ± standard deviation) in the supernatant of PMBC cultures carried out with complete RPMI 1640 alone or with the addition of 5 μg/mL Con A was 0.19 ± 0.26 and 30.08 ± 22.56 ng/mL, respectively. PBMC stimulated with only COS-7 sccaIL-12 SN at 0.025% (1.56 ± 2.31 ng/mL), 0.5% (2.36 ± 3.74 ng/mL), 10% (2.14 ± 2.50 ng/mL) or COS-7 IL-2 SN at 25% (0.52 ± 0.93 ng/mL) produced a greater amount of IFN-γ compared with the cells cultivated with complete RPMI 1640 alone. However, the differences were not significant, probably because only PBMC of 3 or 4 out of 8 dogs and only 1 out of 8 dogs responded to the stimulus of IL-12 and IL-2, respectively (Figure 4). Nevertheless, when PBMC were stimulated with a combination of COS-7 sccaIL-12 SN at either 0.025% (11.34 ± 11.09 ng/mL), 0.5 (13.69 ± 12.62 ng/mL) or 10% (12.73 ± 13.57) and COS-7 caIL-2 SN at 25% or with a combination of COS-7 sccaIL-12 SN at either 0.025 (9,22 ± 10,70 ng/mL) or 0.5% (16,33 ± 16,69 ng/mL) and COS-7 caIL-2 SN at 5%, a significant higher production of IFN-γ was detected (p < 0.05, Dunnett’s test; Figure 5). The combined incitation of IL-12 and IL-2 resulted in the response of 6 or 7 out of 8 dogs.

## Discussion

In the present work, we have reported the construction of plasmids encoding the mature and full-length canine IL-2 for expression in prokaryotic (pRSET-caIL-2) and eukaryotic cells (pcDNA3.1caIL-2), respectively. Comparing the inserts of the constructions pRSET-caIL-2 and pcDNA3.1-caIL-2 with DNA encoding wild-type canine IL-2, previously described by Dunham and collaborators [33], two nucleotide point mutations and complete identity, respectively, was observed. One of the mutations detected in insert of pRSET-caIL-2 was conservative (change to amino acid with similar characteristics) and the other was silent. These mutations have probably occurred due to error in the incorporation of nucleotides carried out by the Taq polymerase used in the PCR reaction [35]. However, it is unlikely that the conservative change of a single amino acid residue would influence too much on the physicochemical characteristics of the protein and interfere with its biological properties. In fact, to determine the impact of such mutation, a tridimensional structure model of canine rcaIL-2-F43L was made using tools of the Swiss Model (http://swissmodel.expasy.org/) and PyMol software (http://www.pymol.org/) and crystallized human IL-2 (pdb IM47) as a reference protein. The tridimensional structure model of rcaIL-2-F43L and human IL-2 showed no significant differences one from another (data not shown).

The pRSET-caIL-2 construction induced the production of rcaIL-2-F43L in the form of inclusion bodies in *E. coli*[36]. In order to refold the rIL-2-F43L, the inclusion bodies were solubilized with a buffer containing urea, the protein was affinity purified and subjected to dialysis to slowly remove the chaotropic agent and allow the formation of the disulfide bridge between the cysteines at positions 59 and 107 of the mature protein (which corresponds to the bridge between amino acid 58 and 105 of the human homologous protein), using a previously described method [31]. This disulfide bridge favors the stabilization of the molecule [33,37,38]. Unfortunately, after being subjected to refolding, the rcaIL-2-F43L showed some degree of instability and a progressive reduction in the concentration of soluble protein in the recombinant interleukin preparation was noted, even when it was stored at -20^o^ C (data not shown). This may have happened by adherence of part of the protein to the tube used for storage or aggregation. Interestingly, the canine IL-2 recently produced in our laboratory using the baculovirus/insect system seemed to be stable during storage at -20°C (unpublished results).

Cerruti-Sola and collaborators [32] have previously reported that the native canine IL-2 was able to induce proliferation of CTLL-2 cells [39], a murine IL-2-dependent CD8^+^ T cell line. In the current work, rcaIL-2F43L or COS-7 caIL-2 SN stimulated the proliferation of CTLL-2 cells in a dose-dependent manner, indicating that the cytokine produced in *E. coli* and COS-7 cells is biologically active. The maximal proliferation induced by rcaIL-2-F43L or COS-7 caIL-2 SN was lower than that promoted by T-STIM. This probably happened due to the fact that, in addition to IL-2, the latter contains other(s) factor(s), including IL-4, also capable of stimulating CTLL-2 cell proliferation [40]. Interestingly, the highest concentration of either rcaIL-2F43L (700 ng/mL), COS-7 caIL-2 SN [50 or 25% (data not shown) and 12.5%] or T-STIM (50%, data not shown) stimulated the proliferation of CTLL-2 cells at a lower level than the maximum. This is probably due to an inhibitory activity of high concentrations of IL-2, or other factors present in the SN, as previously described for human lymphocytes [41].

Several authors have reported that resting NK cells, CD4^+^T lymphocytes, and CD8^+^ T lymphocytes from peripheral blood could proliferate when stimulated with rhuIL-2 or recombinant murine IL-2 [12,42-45]. In fact, resting lymphocytes that constitutively express moderate or high affinity IL-2 receptor, including NK cells, NKT cells, regulatory T cells and CD8^+^ memory T cells (reviewed in [46]), have the potential to proliferate when stimulated *in vitro* without the need of an additional spur.

To determine whether recombinant canine IL-2 could also induce proliferation of resting lymphocytes, PBMC from six healthy dogs were cultured for 4 to 14 days with rcaIL-2-F43L at 50 ng/mL or with COS-7 caIL-2 SN (0.5, 5 or 50%). Indeed, after 6 to 12 days of stimulation, the peripheral blood lymphocytes showed a proliferative response. Previously, Helfand and collaborators, in 1992, reported similar results using rhuIL-2 at 25 U/mL on PBMC of five healthy dogs, showing an increase of proliferation starting by day 5 and peaking by day 8 to 10 of culture [12].

IL-12 stimulates the production of IFN-γ by NK cells and T cells and thereby favors the development of Th1 immune responses (reviewed in [11]). In addition, rhuIL-2 synergizes with IL-12 in the production of IFN-γ in humans [27,28].

To determine the capacity of canine IL-12 and/or canine IL-2 to promote production of IFN-γ, PBMC from eight healthy dogs were cultured with COS-7 sccaIL-12 SN (0.025, 0.5 or 10%) or COS-7 caIL-2 SN (25%) or the combination of COS-7 sccaIL-12 SN (0.025, 0.5 or 10%) and caIL-2 SN (0.2, 1, 5 or 25%). Interestingly, only around half and the minority of the dogs produced IFN-γ after the stimulation with COS-7 sccaIL-12 SN alone or COS-7 caIL-2 SN alone, respectively, in comparison with the COS-7 SN-negative control. However, when COS-7 sccaIL-12 SN (0.025, 0.5 or 10%) was used in combination with COS-7 caIL-2 SN (5 or 25%), PMBC of the majority of dogs synthesized IFN-γ. Such a response to IL-12 and/or IL-2 seems to be very heterogeneous in the population of dogs studied. The data shown herein are in agreement with reports suggesting that IL-2 alone induces the production of IFN-γ in human cells only when used at high concentrations (above 100 U/mL, corresponding to 10 ng/ml) [43,45] and IL-12 by itself promotes the synthesis of low amounts of IFN-γ in lymphocytes [23,27,29,47].

The results described in this study encourage the evaluation of the effects of the administration of rsccaIL-12 and/or rcaIL-2 in dogs, aiming at modulating their immune system, for the development of prophylactic and therapeutic protocols for various diseases. In such studies, low doses of IL-2 and IL-12 could be used in combination, resulting in little toxicity. Those doses alone would be ineffective but, in combination, would promote desirable biological effects [48]. For this, rcaIL-2 produced in the baculovirus/insect cell system (BEV) (unpublished results) and rsccaIL-12 also produced in BEV (manuscript in preparation) could be used. In addition, it would be interesting to determine which are the peripheral blood lymphocyte subpopulations that synthesize interferon gamma when stimulated *in vitro* by the recombinant canine cytokines. This could be achieved by culturing the cells, either in bulk or separated in subpopulations, staining them with anti-cytokine and anti-lymphocyte subpopulation markers fluorescent antibodies and analyzing them by flow cytometry.

An undesirable effect that may result from the use of IL-12 alone or in combination with IL-2, hampering the development of cellular immune responses, would be the expression of IL-10 [49]. Indeed, several authors have reported that treatment with IL-12 and/or IL-2 promotes IL-10 synthesis in T cell clones (Th0, Th1, and Th2), and in peripheral blood cells co-stimulated with anti-CD3 or Con A, in mice [50-52]. Therefore, perhaps, the use of IL-12 and/or IL-2 should be combined with some sort of strategy for blocking IL-10 stimulation, such as the utilization of antagonist peptides, aptamers [53-55], neutralizing antibodies for IL-10 or for the IL-10 receptor (IL-10R1) [56,57], and soluble IL-10 receptor (sIL-10R1) [58,59].

## Conclusions

The results presented herein suggest that the combination of recombinant canine IL-12 and IL-2 can be useful to promote cellular immune responses in dogs.

## Competing interests

The authors declare that they have no competing interests.

## Authors’ contributions

AMP was involved in planning and carrying out some of the experiments (DNA cloning, protein expression and the *ex vivo* and *in vitro* assays), data analysis and in manuscript writing. CGMP was involved in carrying out some of the experiments (*ex vivo* assays and *in vitro* assays) and manuscript writing. LRS was involved in planning and carrying out some of the experiments (DNA cloning, protein expression and the *ex vivo* and *in vitro* assays) and data analysis. NCT was involved in carrying out some of the experiments (*in vitro* assays) and manuscript writing. Y-FC was involved in planning and some of the experiments (DNA cloning). LCPC was involved in planning the study and manuscript writing. GGSO was involved in planning the study and manuscript writing. All authors read and approved the final manuscript.
